# Hospital and Institutional News

**Published:** 1915-09-18

**Authors:** 


### HOSPITAL AND INSTITUTIONAL NEWS.

### AN OLD HOSPITAL REBUILT.

It is gratifying to learn that despite the fact that the building trade in London has practically been at a standstill since the commencement of the war, the new Hospital for Diseases of the Skin in the Blackfriars Road, S.E., is nearly completed, and that in a few short months its work will be resumed in fresh premises, with renewed strength and vigour. Almost since its inauguration the hospital has been in Stamford Street, carrying on the work in what was originally a private house, a relic of South London in the days when this particular district was a residential centre of no mean social standing. The work and the patients have increased, but not so the hospital income in the same proportion, so that there are still urgent calls for financial assistance. The out-patients number close on 6,000 a year, and, although the services at the hospital have been free to the indigent poor, it has long been found necessary to make a small charge to those who can really afford to pay.

### DEATH DANGERS OF DISUNION.

The Local Government Board have been endeavouring to induce the Urban District Councils for the Holme and Colne Valleys to appoint a medical officer for the joint districts, but unfortunately the local authorities have been unable to adopt the suggestion. The two districts adjoin, and it would have been to the advantage of the localities if it could have been expedient to appoint a full-time medical officer, who could have adequately dealt with the health of two large districts, well populated with growing villages, whose inhabitants are principally engaged in the textile industries. The Local Government Board are not adverse to each council appointing its own medical officer, but have expressed the hope that both councils will appoint the same medical man. Meanwhile, the two councils are advertising for applications for the vacant posts, but have so far received none; the small salary offered by each council is insufficient to warrant medical practitioners. If the districts and salaries were combined, the latter might be sufficient to secure a choice from fully qualified medical practitioners.

### PRAISE FOR AN ASYLUM STAFF.

As a general rule there is a tendency to criticise the methods adopted at the various institutions for the mitigation of pain and suffering, and when an opportunity occurs to condemn, it is taken advantage of by every opponent to the institutional treatment of the poor. When praise is meted out to the staff, as is done on very rare occasions, it is all the more gratifying to those engaged in attending to the afflicted. At an inquest at Wandsworth on the body of a young woman who died in the Middlesex County Asylum at Tooting, Mr. Alfred Kimble, her brother, informed the jury that he would like to express his gratitude for the splendid treatment his sister received from the nurses and from Dr. Warwick Smith and the other doctors at the asylum. The care taken, he added, was more than he had seen in any other public institution, and the jury expressed their appreciation of the gratitude expressed to Dr. Smith and the nurses at the asylum. Dr. Smith, who had given evidence, returned thanks, modestly and properly observing that he and the staff were only carrying out their duty.

### THE RETENTION OF A DOCTOR'S FEE.

A somewhat unusual case was heard at the City of London Court, when Mr. William Collins, of Baker Street, sought to recover from Messrs. Ransom and Williams, solicitors, of Devonshire Square, £1 11s. 6d. money received. Plaintiff had a claim against the London County Council for £100, and the defendants acted as his solicitors. He was awarded by the Court £15 15s., including two guineas, the fee due to Dr. Robert Beswick of Bishopsgate, his medical attendant. Plaintiff now sued for £1 11s. 6d., which he said defendants had detained as the doctor's fee. That ought not to be more than 10s. 6d., the amount fixed by the doctor himself. Dr. Beswick informed the Court that his fee was two guineas, and not 10s. 6d., having never told the plaintiff that his charge would be 10s. 6d. Mr. W. Vaughan Williams, for the defendants, stated they had paid two guineas into court out of the money they had received, and this the judge ordered should be paid to the doctor. He marked his sense of the importance of the case by not making any order as to the costs.

### KILLING TWO BIRDS WITH ONE STONE.

Mr. W. J. R. Fox, one of the founders of the Batley and District Hospital has made an interesting gift to that institution. It takes the form of a War Loan investment of the value of £200. The donor has paid for the scrip in full, and has given instructions for it to be registered in the names of the trustees of the hospital, and the payment of interest on the investment to be forwarded in due course to the hospital bankers. By this means the donor not only helps the cause of the hospital but backs up the men at the Front. This is an excellent example of killing two birds with one stone, and we hope others will follow it.

### THE L.G.B., THE GUARDIANS AND ECONOMY.

The Local Government Board circular-letter to local authorities drawing attention to the need of strict economy, to which reference was made in The Hospital of August 25, continues to be discussed at meetings of Boards of Guardians. Despite protests against the circular by individual members, there is undoubtedly a general desire to curtail expenses wherever possible. In various places changes in the dietary have been resolved upon, and provided that the medical officers are satisfied that such changes will not impair the efficiency of the institution there would appear to be nothing objectionable in this policy, especially if considerable saving can be effected. Economies in various other directions have been proposed. Thus the Kendal Guardians have been considering whether they could not save something on the cost of their account books. This is essentially a question for the accountant. But the statement at a meeting of the Guardians that books costing £2 each and ruled with forty different columns can be replaced without disadvantage by sixpenny memorandum books, can hardly be one of knowledge or practice.

### A MENTAL HOSPITAL SUPERINTENDENT AND THE WAR.

The difficulty of obtaining medical superintendents for mental hospitals has been brought prominently to notice at Dundee, where the General Board of Control of the West Green institution is hesitating to allow Dr. Tuach-Mackenzie to go on active service because of the obstacles in the way of making satisfactory arrangements for filling the vacancy. The medical superintendent has been asked by the War Office if he is willing to accept service with troops in Egypt as a specialist in nervous diseases, and he has agreed to undertake the duties, provided he receives the sanction of the Board. The Board, while recognising that the Army has first claim, is naturally anxious that the place of the medical superintendent should be filled during Dr. Tuach-Mackenzie's absence, and in the hopes of finding a solution of the problem a special committee has been appointed to consider it. Mental hospitals have perhaps suffered less, at any rate as regards their principal medical officers, than general hospitals by the absorption of medical men in the Army; but this case serves to show how the shortage of practitioners is being felt in special hospitals.

### OPERATIONS ON NON-PAUPERS AT POOR-LAW INFIRMARIES.

At their last meeting the Oldham Guardians decided by a large majority to open their surgical wards to paying patients. A committee was appointed to consider applications from persons requiring operative treatment who could not afford the fees of private nursing homes and for whom there were no vacancies available at the Oldham Infirmary. It was agreed that in urgent cases the applicants should be admitted forthwith and the circumstances investigated afterwards. This is an important advance in the activities of Poor-Law Guardians, and it will be interesting to see how the Local Government Board views the matter. It is common enough, of course, for patients who are not paupers to be admitted to Poor-Law infirmaries for surgical operations, especially in cases of urgency. This is the first time, however, that a Board of Guardians has practically invited ratepayers to make use of the facilities for surgical treatment provided by the rates. If the Local Government Board allows the scheme to proceed other Boards will certainly follow the example of Oldham, and the result will be far-reaching and beneficent changes in Poor-Law administration.

### AN ELECTROPHONE BULLET PROBE.

We understand that the War Office has sent ten electrophone bullet probes to the Dardanelles. This instrument was designed by Dr. Charles Firth, F.R.C.S., consulting surgeon to Gravesend Hospital. According to a description in the *Gravesend and Northfleet Standard*, it consists of a silver tube, 8 in. or 9 in. long, of the size of a No. 3 catheter, lined with indiarubber, and a central wire or core. The tube is connected with one terminal of an electric bell and battery combined, and the core with the other terminal. When the tube and core both touch the bullet, or any metal body, the bell rings. The core is freely movable within the rubber-lined tube, so that it can be made to project about 1 in. at the lower end. This enables a bullet to be discovered when the tube is not end-on to it, but passing it on one side of a sinus; then, by projecting the core and slowly withdrawing the probe, one can get a ring at the moment they both touch the bullet. In this way it is often possible to locate the position of a metal foreign body. If the claims made for this ingenious contrivance are substantiated, there may be available for surgeons a simple instrument which has the advantage of being readily attached to a source of electricity almost universal.

### WHY ARE WORKMEN'S SUBSCRIPTIONS WITHHELD?

In The Hospital of September 4 we alluded to the falling-off in the workmen's subscriptions to the Merthyr General Hospital. In this instance the decline appears to have been due not to lack of benevolence on the part of the workmen of the district, the withholding of subscriptions being their method of expressing a specific grievance. But diminished contributions are reported from other districts where the workmen have apparently no excuse for the neglect of their charitable duties. Thus, presiding at the annual meeting of Corbett Hospital last week, Viscount Cobham remarked that it was somewhat disquieting to see the large falling-off in the past two years in the workpeople's contributions; and speaking at the annual meeting of the subscribers to the Dunoon Convalescent Homes, Lord Inverclyde deplored the fact that the subscriptions from the workmen had declined. It is difficult to understand why there should be such a display of withheld support at a time when the working classes are being paid higher wages than they have ever been paid before. The deficiency may to some extent be caused by the fact that some former contributors have joined the Colours, but it is surely the privilege of the workmen who remain at home to make this deficiency good. They can well afford to do so. Have they some genuine cause for their action in these cases?

### AN UNREASONABLE BOARD OF GUARDIANS.

At the last meeting of the Ballymena Guardians, the County Antrim Infirmary was the subject of adverse comments which appear to be unjustified. The Guardians sent two patients suffering from cataract to the infirmary for treatment. The ophthalmic surgeon found they would not be ready for operation for three months and suggested that at the end of that time they should be sent to the Belfast Ophthalmic Hospital as the County Infirmary did not possess facilities for dealing with cataract cases. When this suggestion was placed before the Board several Guardians asserted that the infirmary was not an up-to-date institution as it could not treat cataract cases, and a resolution was passed asking the County Council to vote against the annual contribution to the infirmary. The Guardians are evidently ignorant of the complexity of methods of treatment nowadays. It is only under exceptional circumstances that a hospital can be equipped fully in every department. A hospital may be thoroughly up to date and well able to deal with all ordinary cases of sickness, and yet not possess the requirements for some of the specialised forms of treatment. It is an unnecessary extravagance to have expensive apparatus at every hospital for the treatment of the rarer forms of disease, when one installation can deal with all the cases occurring within a given area. It is strange that this common-sense view did not occur to the Guardians.

### LOCAL GOVERNMENT IN SCOTLAND—TWENTY-FIVE YEARS' PROGRESS.

At the forty-first Annual Congress of the Incorporated Sanitary Association of Scotland, Mr. Robert Lambie, the President, spoke on "Twenty-five Years of Local Government." He pointed out that the Local Government (Scotland) Act of 1889 inaugurated a new era as regards the public health services. Medical and sanitary officers found a place in local affairs they had never before occupied, more especially in the county areas. Water supplies, drainage schemes, housing improvements, lighting, scavenging, public libraries, and the like had all come on apace. The average death-rate had fallen from 19 to 15 per 1,000; the infantile mortality from 121.3 to 105.5 per 1,000 births; and the phthisis death-rate from 1.76 to 1.12 per 1,000. Roads and footpaths had been improved, pure and sufficient water supplies had been provided, foul and unsightly ditches and ash-pits were to a great extent things of the past, food and milk supplies were strictly supervised and controlled. Passing to the future, he said it was evident great work still lay before them. A local authority ought not to be allowed to neglect in any way its public health and sanitary duties owing to the fear of high rating. National services such as the public health ought to be so recognised by the Imperial Government that a uniformly high standard would be assured in every town and district in the country, with a burden in respect of rating as nearly as possible uniform.

### THE NOTIFICATION OF MEASLES.

At the same meeting a paper was read by Dr. A. Campbell Munro, the county medical officer of Renfrewshire, on "The Care of the Health of the Child." He said that the stand which the Central European Powers had been able to make was largely due to their high birth-rate. Our diminishing birth-rate had been one of the most disconcerting factors in the decline from power as a nation as compared with the Germanic countries. He hoped that in the coming years the birth-rate would show an increase. However that might be, it was important to take every step to safeguard the health of the children who were born. The first element in a scheme of child welfare was the appointment of a health visitor. Whooping-cough and measles took a heavy toll of life in the period between infancy and school age. He felt that for the most part local authorities were doing nothing to stay that waste of life. Nothing could be done without knowledge of the existence and whereabouts of the cases, and he advocated compulsory notification of the diseases. A resolution was proposed that measles and whooping-cough should be made compulsorily notifiable, but was defeated by a large majority. The result is disappointing. The death-rate from measles and whooping-cough in children is greater than that of all the other zymotic diseases put together, and, as Dr. Munro pointed out, preventive measures can only be efficiently employed when there is full knowledge of the whereabouts of the cases.

### INTERFERING BETWEEN PRACTITIONER AND PATIENT.

A Devonshire tuberculosis officer has addressed a complaint to the Sanatorium Sub-Committee of the Insurance Committee to the effect that he has been much annoyed since he took up his duties by his patients being visited and questioned, apparently by members of a District Committee, as to whether they were satisfied with the medical treatment. The letter, which is none too strongly worded for the occasion, goes on to say: "You will never get high-class men, or men with high qualifications, with perfectly independent judgment, if that sort of espionage is permitted. We have quite difficulties enough to exercise a right judgment in all things, and to be quite fair to all parties without this extra stimulus of finding fault with our work. It is in our power to do an endless amount of harm, and an absolutely independent judgment is essential to good, honest work. A flagrant instance of this interference has come to my knowledge in this town quite recently." It is to be hoped that this timely protest will produce a good effect. There can be no possible objection to members of District Committees inquiring after the health of patients who are undergoing treatment under the administration of the After-Care Committee, but the notion that membership of a committee gives a man or a woman the right to interfere between doctor and patient is preposterous. District Insurance Committees appear to be of little value under any circumstances, and if one of their habits becomes that of meddling with other people's business it is about time they ceased to exist.

### A PANEL DOCTOR AND HIS PATIENTS.

It must be apparent to everybody that the difficulty of giving proper attention to a large panel of insured persons and a considerable private practice at the same time is almost overwhelming; and so long as panel doctors persist in attempting to perform this feat so long will trouble arise. At Cambridge the other day a committee of inquiry appointed by the Insurance Commissioners sat to investigate complaints made by the local Insurance Committee relating to the conduct of a Cambridge panel practitioner. It was alleged that on various occasions insured persons had been attended not by the practitioner, but by his dispenser, who had written prescriptions and supplied medical certificates. The practitioner admitted that there had been certain irregularities, but urged that these had been committed inadvertently. Now when we consider that the panel doctor in question had over two thousand insured persons on his list and that last year he saw 12,457 panel patients, and that with private patients the total was brought up to 20,660 consultations and visits, it is obvious that he was overworked. But a National Health Insurance practitioner is not compelled to have too big a panel, and it is surely his duty to put a check on the growth of his list. Concerning this particular case the Commissioners will give their decision in due course, but whatever the decision may be, it is to be hoped that other panel doctors will keep their list of patients within reasonable limits.

### GREAT GIFT FOR CARDIFF HOSPITAL.

At a meeting of the board of management of King Edward VII.'s Hospital at Cardiff on Wednesday, last week, a generous gift of £5,000 by an anonymous donor for the erection of a maternity flat was announced. This magnificent sum is to defray the cost of a flat to be built over the Bute wards, which will comprise a ward of sixteen beds, a small emergency ward for two beds, a labour theatre with every essential appointment, examination rooms, the various baths, and everything necessary to make the flat as perfect as possible for its important purpose. The need for maternity wards at the Cardiff hospital has long been urgent, and it is an especial cause for congratulation that the ambition of the board of management has been achieved at this time of strain and stress. When we consider that the maternal mortality rate for South Wales is much higher than that for England and Wales, and something like 40 per cent. higher than the London rate, it becomes apparent what a humane object this addition to the Cardiff hospital is capable of serving. As Colonel Bruce Vaughan eloquently observed, in expressing the thanks of the board to the anonymous donor, the advent of the maternity flat means "holding out the sympathetic hand of succour and help and of scientific skill to at least 450 mothers in their hour of trial and to 450 children every year." It also means that facilities will be afforded to students for maternity training under supervision within the precincts of the hospital, and so obviate the necessity for seeking instruction elsewhere. This anonymous and magnificent gift will serve as an eloquent appeal to others and as a stimulating example of the highest form of philanthropy.

### SANITARY PROBLEMS IN HOSPITALS FOR INDIANS.

Like most other organisations which hold annual Congresses, the Royal Sanitary Institute was compelled to change its plans for this year's meeting. Arrangements had been made for a visit to Newcastle-on-Tyne, but these had to be abandoned, and instead a provincial sessional meeting was held at Brighton on September 3 and 4. There are so many of the problems arising out of the war which involve questions which come within the purview of the Institute that it would have been a misfortune if an opportunity had not been taken for the discussion of such matters. A remarkably interesting paper on "Sanitary Problems in Hospitals for Indian Troops in England" was communicated by Major S. P. James, M.D., I.M.S., who described in considerable detail the transformation of the Brighton Workhouse into a hospital for the accommodation of two thousand wounded Indian soldiers. This hospital is conducted by officers of the Indian Medical Service, with a staff of assistant surgeons and sub-assistant surgeons belonging to the Indian Subordinate Medical Department and a *personnel* numbering about six hundred. The problem before the management was to convert ordinary buildings to hospital buildings, so as to enable the Indian patients and staff to live in accordance with the customs of their particular class and according to the rules enjoined by their religious sects. The structural problem, however, was not the most important one from the point of view of the members of the Institute, and Major James drew particular attention to the arrangements which had to be made for enabling Indians to live in accordance with the customs of their country.

### HOW CASTE PREJUDICES WERE MET.

In the first instance "local colour" was obtained by the introduction of wounded soldiers of different classes, whose racial characters were explained by the lecturer. All these classes have customs and prejudices differing from those of the other classes, which have to be strictly adhered to and safeguarded in order to prevent the individual members from losing caste. At Brighton this has been ensured by appointing a committee composed of Indian officers and non-commissioned officers of every caste. Some idea of the difficulty encountered in meeting all prejudices may be gathered from the fact that as a routine measure eight different kinds of diet are arranged for and separate cookhouses are provided for six different classes; and as regards drinking water, separate drinking fountains are furnished throughout the hospital for Mohammedans and Hindus. Ample facilities are provided for washing and bathing, and there are no fewer than seventy specially constructed bathrooms in the hospital, each accommodating several patients at a time, and having hot and cold water supplies laid on to overhead showers. Major James reminded his audience that an Indian must bathe the greater part of his body before each time of prayer, and that a good Mohammedan must pray five times daily; it is therefore not surprising to learn that the amount of water used in the hospital averages between sixty and seventy gallons per head per day. Another interesting part of the address from the point of view of the sanitary engineer was that dealing with the construction of the closets so as to meet the difficulties created by the peculiar methods of the patients. For the speedy and efficient manner in which all the obstacles have been overcome great praise is due to those whose task it was to organise this great war hospital.

### ANOTHER DRAIN ON THE FUNDS.

A question that is no doubt perplexing those who are responsible for the purchase of drugs for public institutions is whether it would be advisable to buy the winter's supply of cod-liver oil at once, or to wait a little longer in the hope that prices will be lower. At the present time the value of cod-liver oil is extremely high, and if purchases have to be made at the quotations now ruling the drug bill will be seriously affected, since prices are at least four times an average figure. The result of last season's Norwegian fishing was a fairly good yield of oil, and had the circumstances been normal there would probably have been no difficulty in procuring supplies at a quarter the present quotation. But although the yield was about an average one, the demand has been unprecedented, Germany and Russia having bought in such enormous quantities that the stocks of cod-liver oil remaining in Norway are probably not much more than one-fifth the size of the stocks that would have been otherwise available. Moreover, there appears to be no doubt that these small stocks are concentrated in the hands of strong holders who are not likely to give way unless extraordinary pressure is brought to bear on them. In view of the fact that the chief consuming season is close at hand, and that supplies will have to be obtained at whatever cost, it does not seem at all improbable that holders will be able to get their own way. In fact, a small business has been done this week at a price very close to the exorbitantly high figure that has been nominally quoted for some time. Looking at the matter from all standpoints, there would appear to be little hope of any substantial reduction in price, and under the circumstances there is perhaps little to be gained by postponing the making of purchases.

### HOSPITALS AND THE SPIRIT TAX.

The reassembly of Parliament serves as a reminder of the promise of the Chancellor of the Exchequer to introduce in the House at an early opportunity a Bill to provide for the use by hospitals of duty-free spirit in the manufacture of medicinal preparations. It will be remembered that, mainly owing to the opposition engineered by the British Medical Association and the Pharmaceutical Society, a clause which it was proposed to insert in the Finance Bill to provide for this concession—which would have resulted in a saving to the hospitals of Great Britain of something like £30,000 a year—was abandoned by the Government. Mr. McKenna promised, however, that at an early date a clause embodying a similar principle would be introduced in another Bill, and he implied that facilities would be given for passing the Bill, this promise being conditional upon the absence of opposition. As we showed on a previous occasion, the only opposition possible, apart from that for which the British Medical Association and the Pharmaceutical Society were directly responsible, could arise out of the misconceptions of the Temperance party. And as the misconceptions of that party are based chiefly upon the representations made in the House of Commons on behalf of the Association, it is obviously the duty of the Association to take steps to remove them. We anxiously await the introduction of the promised Bill, and if its terms are less generous than those voluntarily offered by the Government three months ago, we shall have something to say about the tactics of the British Medical Association. The effect of the original Government proposal would have been equivalent to a legacy of three-quarters of a million sterling to hospitals, and we are not likely to forget the part the British Medical Association played in delaying or destroying this bequest.

### HOSPITALS ON WHEELS.

Space would not permit to give even brief details of the various hospital trains that have been completed in recent weeks, but it is worthy of note that what is said to be the finest ambulance train in the world has been constructed for use in France by the Caledonian Railway Company. The train was formally opened at the beginning of this month by the Duchess of Montrose, and has been built by order of the Government at the Company's St. Rollox Works. It consists of sixteen corridor carriages, is 300 yards in length, weighs 442 tons, and has accommodation for 454 patients and 44 attendants—a total of almost 500 persons. The carriages are equipped with all the most modern contrivances for the comfortable conveyance of the wounded, and the train has cost £20,000. Another train which is worthy of special note is the Great Eastern ambulance train, which has also gone to France. This has five ward cars for lying-down cases with thirty-six cots apiece; five cars for sitting-up cases with fifty-six cots each; a pharmacy car; and a treatment room equipped with an operating table, etc. The staff car suffices to accommodate four doctors and nurses, and there is a car capable of sleeping twenty-eight orderlies. In addition, there are two kitchen cars with accommodation for cooks and stores, a mess room, and two brake vans—one with a section for stores, while the other constitutes a ward for infectious cases. Something like 2,000 gallons of water can be carried on the train, and nearly every car has a tank for drinking water. Special sanitary arrangements are provided throughout the train, and one of the lavatories is fitted with a shower-bath. We have not yet seen the specification of the train constructed by the Caledonian Railway Company, but if it is deserving to be called the finest ambulance train in the world we think the Great Eastern train could safely be described as the next best. Incidentally, it is worthy of note that before it was shipped to France this latter train was inspected by over fifty thousand people, who paid sixpence each for the privilege, the result being that a sum of over thirteen hundred pounds was handed over to a fund for providing comforts for the railway troops who have gone to join the Army in France.

### MEDICO-LEGAL DANGERS OF SILVER NITRATE.

THE *Ophthalmoscope* records a case, reported from France, showing the risks which attend the use of silver nitrate in conjunctivitis. The patient had long suffered from chronic conjunctivitis and had been treated unsuccessfully by various methods. His medical adviser then proposed the use of solid nitrate of silver, and this was adopted. The caustic was applied to the whole of the palpebral conjunctiva, and afterwards free lavage with a solution of sodium chloride was practised. This was followed by much suffering, and examination showed the conjunctivæ to be chemosed and turgid and the corneæ opaque. Vision was seriously compromised, and the patient suffered from headaches and neuralgia. He brought an action against his doctor, and was awarded £800 damages.

### EYE DEFECTS AND THE FIGHTING LINE.

IN a recent discussion, reported in the *Ophthalmoscope*, Mr. Richardson Cross suggested that men who have faulty right eyes, but can see well with the left eye, should be accepted for the Army, but should be placed in a special squad. From the Director-General he had received the message, "Do not send us any cases of nystagmus." It had been found that men with nystagmus not only shot badly, but also to one side, so that they may injure their comrades rather than the enemy. Mr. T. H. Townsend proposed that these men should be detailed for the "donkey work," which claims nearly seventy soldiers in each regiment. In this way they could still be of service to their country.

### AN EPISODE IN THE HISTORY OF THE SOCIETY OF APOTHECARIES.

THE partial demolition of the Old Mill House, belonging to the Apothecaries Society in Water Lane in the City of London, has recently been the subject of many references in the lay Press, and the occasion has been utilised to recall events associated with the early history of this Society. But we have not seen anywhere such a concise account of the foundation of this Society as that which is recorded in a book published in 1704, under the title "Short Answers to Tentamen Medicinale." "'Tis very well known," says this authority, "there was no such thing as a company of Apothecaries in the beginning of King James I. reign, but what drugs and medicines were then in use, were sold in common by the grocers; and as for the preparation and compounding of them, that the physicians principally took care of themselves. But this growing too servile and laborious a business, and no other means being likely to be found out for easing themselves of it, but by lopping off a considerable number of grocers who had mostly been brought up that way, and constituting them a company by themselves, wholly to be employed in the business of pharmacy, in selling of drugs and preparing and compounding medicines, according to the Physicians' orders and directions; in order to do this they obtained a charter for them to the number of a hundred and fourteen." The charter referred to was granted in 1617, and the power of searching the shops of apothecaries within seven miles of the City of London and examining their drugs was vested in the Society. Of the history of the Society since that date books could be written; indeed books have been written. But perhaps the most interesting records are those which relate to the quarrels between the apothecaries and the physicians, in which the wits on neither side spared their efforts. The apothecaries on their side complained of the growing practice among physicians of dispensing their own medicines, and to this complaint the reply was that "the physician was not obliged to prostitute his honour and conscience by overloading his patient, to oblige a craving apothecary, or run the risk of being undermined in his reputation by slanderous suggestions, for not submitting to be the apothecaries' under-pickpocket." Naturally the apothecaries were not silent under this abuse, and one of their number published a reply some idea of the character of which can be gathered from the retort that "when a Physician has got a guinea for his visit, it seldom much concerns his honour or conscience, how the apothecary shall get a shilling for his medicines." Many pamphlets were published on both sides, and copies of them are much sought after by lovers of old books of their kind.

### THIS WEEK'S DRUG MARKET.

OWING to the extreme prices quoted for practically all the synthetic drugs, business in these products is being restricted to the smallest possible requirements of purchasers, and few are buying more than is necessary for immediate use. While this appears to be a reasonable policy, there are no indications that any reduction is likely to take place for some time to come. Since our last report was written, acetyl-salicylic acid, guaiacol carbonate, phenacetin, and sulphonal are again dearer. The value of opium, which has been practically unchanged for some time, now shows an advancing tendency, but up to the present the advance has only been slight; the quotations for morphine and codeine are firmly maintained, and makers continue to be extremely busy. Quinine still has an upward tendency in price, and a fair amount of business is passing; it would probably be a wise thing to replenish stocks of this drug at an early date. Quicksilver is again slightly cheaper, but mercurials are unaltered. At the recent public sale of drugs in Mincing Lane, Cape aloes, cubebs, cardamoms, honey, and Japanese refined camphor sold at somewhat lower rates, but menthol and ipecacuanha realised rather higher prices.

